# Duplication cyst in adult cases: a journey from diagnosis to treatment

**DOI:** 10.1093/jscr/rjae460

**Published:** 2024-07-13

**Authors:** Nang Van Pham, Doi Van Mai, Phu Diep Thien Duong, Huan Hoang Lam, Hung Huynh Vinh Ly, Luan Van Nguyen

**Affiliations:** Department of General Surgery, Faculty of Medicine, Can Tho University of Medicine and Pharmacy, 179 Nguyen Van Cu Street, An Khanh ward, Ninh Kieu district, Can Tho 900000, Viet Nam; Department of General Surgery, Faculty of Medicine, Can Tho University of Medicine and Pharmacy, 179 Nguyen Van Cu Street, An Khanh ward, Ninh Kieu district, Can Tho 900000, Viet Nam; Department of General Surgery, Faculty of Medicine, Can Tho University of Medicine and Pharmacy, 179 Nguyen Van Cu Street, An Khanh ward, Ninh Kieu district, Can Tho 900000, Viet Nam; Department of General Surgery, Faculty of Medicine, Can Tho University of Medicine and Pharmacy, 179 Nguyen Van Cu Street, An Khanh ward, Ninh Kieu district, Can Tho 900000, Viet Nam; Department of General Surgery, Faculty of Medicine, Can Tho University of Medicine and Pharmacy, 179 Nguyen Van Cu Street, An Khanh ward, Ninh Kieu district, Can Tho 900000, Viet Nam; Department of Pathology, Can Tho University of Medicine and Pharmacy, 179 Nguyen Van Cu Street, An Khanh ward, Ninh Kieu district, Can Tho 900000, Viet Nam

**Keywords:** duplication cysts, gastrointestinal tumor, ileal duplication cysts, colonic duplication cysts

## Abstract

Duplication cysts are rare congenital abnormalities of the alimentary tract, typically manifesting symptoms in the first 2 years but uncommon in adults. Medical data on duplication cysts is scarce in Vietnam’s Mekong Delta region. These two adult cases aim to provide fundamental knowledge, clinical characteristics, diagnosis, risks, complications, surgical and observational treatment methods, and future bilateral tumor research. Case 1: A 21-year-old male with intestinal obstruction symptoms. Computed tomography (CT)-scan revealed a strangulated small bowel obstruction with ischemia. Laparotomy discovered a twisted ileal duplication cyst causing necrosis in ~30 cm of the small intestine. Case 2: A 34-year-old woman hospitalized for right lower quadrant pain. CT-scan showed a cystic structure protruding into the ascending colon lumen. She underwent a laparoscopic right hemicolectomy, and an ascending colonic cyst was found in the specimen. Conclusions: Duplication cysts are rare anomalies, especially in adults. Comprehending and acquiring knowledge ensures prompt diagnosis and appropriate treatment.

## Introduction

Gastrointestinal tract duplication cysts, also known as enteric duplication cysts, are rare congenital abnormalities that can appear in different parts of the body, but they most commonly occur in the gastrointestinal tract. These cysts develop between the fourth and eighth weeks of fetal development [1]*.* It is worth noting that gastrointestinal tract duplication cysts occur in ~1 in every 4500 births, with the ileum being the most frequent location (60%), followed by the jejunum and duodenum [1, 2]. Although they are generally considered benign conditions and symptoms usually arise by the age of 2, such as abdominal pain, nausea, vomiting, or bleeding, etc. [1, 3, 4]. However, there have been rare cases where symptoms appear later in adulthood, which carries a potential risk of malignancy (5%–6%), such as adenocarcinoma, squamous cell carcinoma, gastrointestinal stromal tumors, and neuroendocrine tumors [1, 3, 5],

The diagnostic workup typically begins with an ultrasound which is considered the preferred imaging method because it is a simple, readily available diagnostic imaging technique and can effectively show both the double wall sign and the presence of peristalsis. This is a non-invasive tool that plays a crucial role, especially in emergency cases involving children under 2 years old [6]. Computed tomography (CT) scans, which are more sensitive, can not only provide information about the location and size of the cyst but also detect any complications, including volvulus, intussusception, obstruction, etc., associated abnormalities, and the anatomical relationship with nearby structures [7]. Recent studies emphasize the use of Endoscopic Ultrasound-Guided Fine Needle Aspiration (EUS-FNA) for diagnosing cysts, as it is crucial in obtaining a definitive diagnosis and ruling out more serious conditions [8].

Surgery is the preferred treatment for most symptomatic or complicated cases [8, 9]. The decision on surgical resection in asymptomatic patients remains a topic of debate. Some authors advocate for the routine resection of asymptomatic colonic duplication cysts, citing concerns about potential malignant degeneration (67%) [1, 3, 5].

In contemporary Vietnam, there is a notable scarcity of reports on the diagnosis and treatment of duplication cysts. This condition is deemed rare in adults, often discovered serendipitously or when manifesting symptoms and complications. The accurate diagnosis of duplication cysts and the crucial task of ruling out malignancy prior to surgery pose significant challenges. Consequently, this study aims to showcase typical cases, contributing valuable insights to enhance understanding and facilitate timely diagnosis and treatment within the realm of digestive surgery in Vietnam.

## Cases series

### Case 1

**Figure 1:**
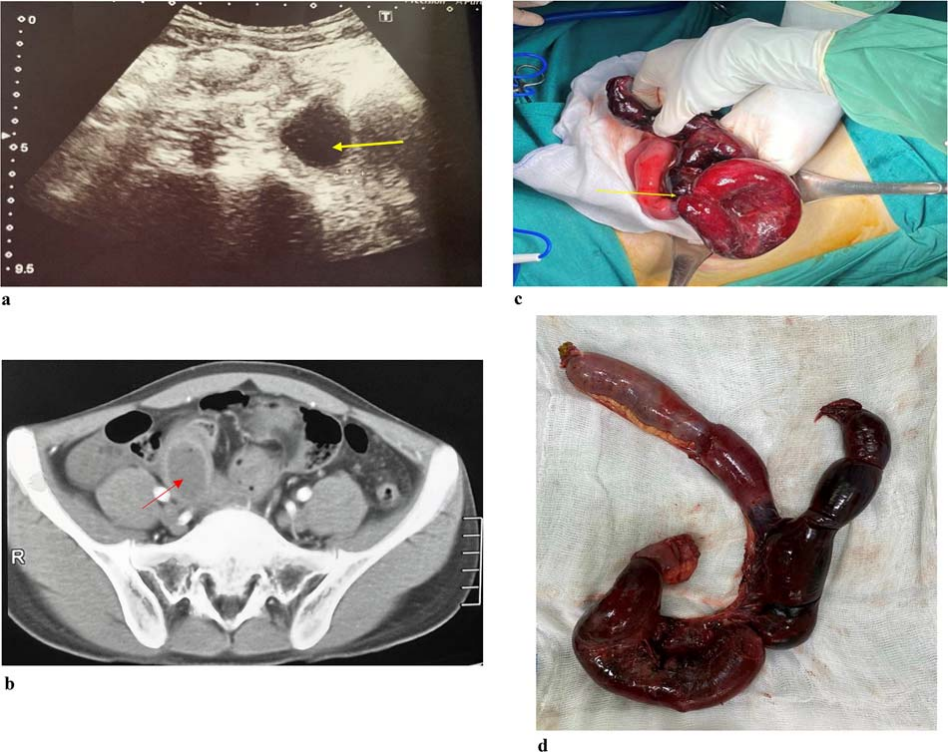
(a). Ultrasound shows an arrow pointing to anechoic structure with hypoechoic wall. (b) Axial CT scan showing an arrow pointing to an ileal cyst dilation with bowel ischaemia. (c) Intraoperative image of small bowel ischaemia with an arrow pointing to the strangulated location. (d) Postoperative image shows ileal duplication cyst.

A 21-year-old male patient presented to the emergency department due to colicky right iliac fossa pain with progression to distension, nausea, vomiting, and obstipation. After 72 hours of attempting self-medication at home without any improvement, his family decided to take him to the hospital. During the physical examination, the patient exhibited a body temperature of 39°C, and his tachycardia was 112/min, and blood pressure was 100/70 mmHg. Abdominal ultrasound (Fig. 1) was reported as the right iliac region has an intestinal structure ~81 × 31 mm in size, with a wall thickness of 9 mm and continuous with an anechoic cyst-like structure ~29 × 24 mm in size, with a hypoechoic wall ~5 mm thick. There is little fluid around. CT scan (Fig. 1b) revealed thickening of the small intestinal wall and ileal cyst dilation with bowel ischaemia. Based on the clinical and radiographic findings, he was diagnosed with strangulated bowel obstruction. The patient proceeded to have an explorative laparotomy performed. Intraoperative findings indicated that an isolated tubular ileal duplication had become twisted, resulting in necrosis of the adjacent small intestine extending ~30 cm (Fig. 1c). Surgical intervention involved performing a segmental resection of the bowel and cyst, followed by reconstruction using a side-to-side anastomosis technique. On histopathological examination (Fig. 1d) showed a separation of the ileal duplication cyst. The inner lining of the cyst revealed mucinous epithelium with mucin. He had a successful recovery and was discharged after 7 days.

### Case 2

**Figure 2:**
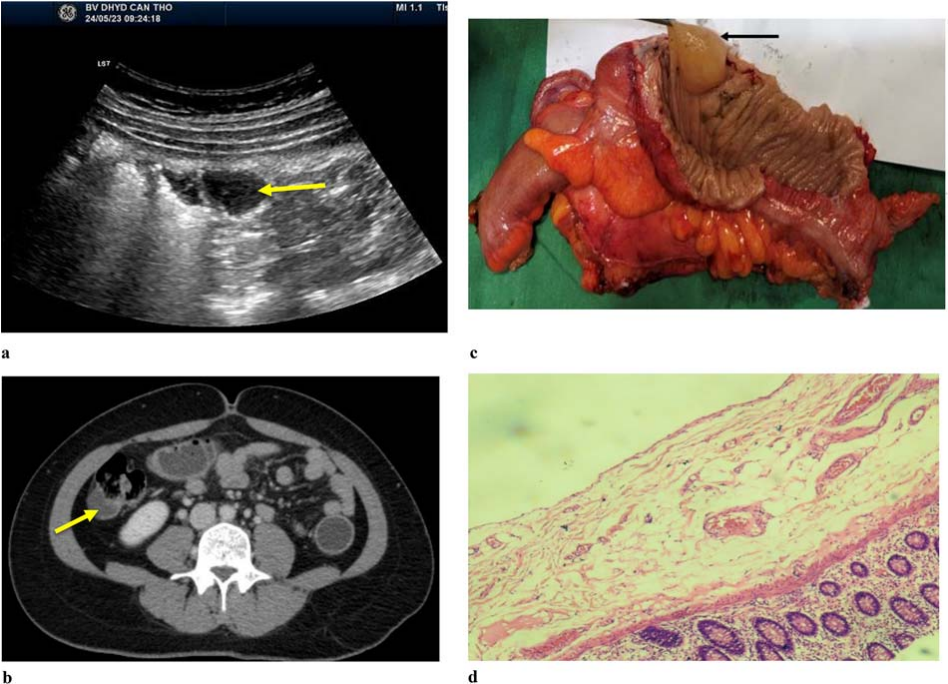
(a) Abdominal ulstrasound shows an arrow pointing to anechoic structure with septation. (b) Axial CT scan showing an arrow pointing to a cystic structure protruding into the lumen of ascending colon. (c) Arrow pointing to colonic duplication cyst. (d) Microscopic examination, the inner lining of the cyst consisted of flat epithelium containing mucin.

A 34-year-old female patient presented to the hospital due to a one-year history of right lower quadrant (RLQ) pain. There were no reported symptoms of weight loss or changes in bowel habits. She had no relevant medical or surgical history, and no family members had a history of gastrointestinal cancer. Physical examination revealed mild tenderness in the RLQ but was otherwise unremarkable. Abdominal ultrasound (Fig. 2) showed the right lumbar cystic lesion, adjacent to the ascending colon, the lesion appeared as a non-adhesive, anechoic structure with septations and no Doppler pattern. The size was ~35 × 17 × 26 mm. CT scan (Fig. 2b) demonstrated a cystic structure protruding into the lumen of the ascending colon, clearly margin, enhancing wall with a size was ~24 × 15 mm. A subsequent colonoscopy did not detect lesions. Based on the clinical and radiographic findings, she was diagnosed with a symptomatic colonic duplication cyst. Intraoperatively, an ascending paracolic cyst was found. The patient underwent laparoscopic right hemicolectomy, and she made an uneventful recovery and was discharged on the seventh day after the operation.

The macroscopic examination of the histopathology sample (Fig. 2c) identified a 20 mm cystic mass located adjacent to the ascending colon. Upon microscopic examination (Fig. 2d), it was observed that the inner lining of the cyst consisted of flat epithelium containing mucin.

## Discussion

Gastrointestinal duplication cyst is an infrequent congenital anomaly, occurring in ~1 in 4500 births, and it tends to be more prevalent in males [2]. These cysts can be classified into two types based on their structure: cystic (80%) and tubular (20%) [8]. While gastrointestinal duplication cysts can occur anywhere from the oral cavity to the anus, they are most commonly found in the abdominal cavity (75%) [10]. Among the gastrointestinal duplication cysts, the ileum is the most commonly involved site, accounting for 60% of cases. On the other hand, duplication cysts in the large bowel are rare, comprising only 6.8% [8].

Duplication cysts can be asymptomatic or symptomatic, with the manifestations depending on the type and location of the anomaly, classified as foregut, midgut, or hindgut. Cysts in the oral cavity or esophagus may cause respiratory distress or dysphagia, while large, rapidly growing cysts can lead to chest pain behind the sternum, hemoptysis, and infections. Gastric or intestinal duplications may produce nausea, vomiting, abdominal bloating, or a palpable mass. Recurrent abdominal pain is a common symptom due to high cyst pressure from secretion buildup [1, 3, 4, 9]. Therefore, there are no specific clinical signs for duplication cysts, and imaging modalities need to be combined for a more accurate diagnosis.

The initial diagnostic workup involves abdominal sonography. Ultrasound is the preferred imaging modality, primarily for its capacity to detect the double wall sign and peristalsis. Peristalsis in duplication cysts often presents as ring contractions, accompanied by a concentric contraction pattern of the cystic wall. Notably, the presence of peristalsis in a juxta-enteric cyst is highly indicative of a duplication cyst and can serve as a valuable diagnostic feature [8, 11]. While CT scans provide valuable information about the location, size, and complications of the cyst, as well as any associated anomalies and its anatomical relationship with nearby structures [7]. In Case 1, the CT demonstrated small bowel obstruction with ischemia due to torsion of the ileal duplication cyst, prompting emergent surgical exploration. Certain endoscopic techniques like colonoscopy can aid in the diagnosis if the duplication cyst communicates with the gastrointestinal lumen or causes extrinsic significant compression. However, as seen in Case 2, non-communicating colonic duplications may be missed on colonoscopy. Therefore, it is necessary to combine it with other imaging modalities to avoid missing any lesions. Although, based on numerous literary sources, it is widely acknowledged that abdominal ultrasound, CT scans, and endoscopy play a crucial role in the diagnosis and differentiation of gastrointestinal tumors from other conditions [5, 9, 12]. However, the drawback of these tools is their inability to accurately assess the benign or malignant nature of the cyst. Especially, in asymptomatic patients, the decision regarding surgical resection is a matter of debate. While some authors argue for resection due to the potential risk of malignant degeneration of the duplication cyst, others recommend observation. Therefore, EUS-FNA is a tool that can aid in obtaining biopsy samples from the cyst for histopathological examination, thereby informing treatment decisions [8].

The main risk associated with gastrointestinal duplication cysts is their potential for malignant transformation. Overall, the reported risk of malignancy in these cysts is 5%–6% [4, 13]. However, this risk is significantly higher for duplication cysts located in the colon; with studies indicating up to 67% of colonic duplications may undergo malignant degeneration [3, 14]. While adenocarcinoma remains the most common malignancy found in duplication cysts, there have been rare reports of other types of cancers such as squamous cell carcinoma, gastrointestinal stromal tumors, and neuroendocrine tumors [1]. This considerable malignancy risk, particularly in colonic duplications, is an important factor driving the debate around routine surgical resection versus observation in asymptomatic cases. In addition to the malignancy risk, duplication cysts can also leads to various complications [9]. One major complication is intestinal obstruction, as illustrated in Case 1 where a twisted ileal duplication cyst caused a strangulated small bowel obstruction in a 21-year-old male patient. This obstruction can progress to a life-threatening complication of bowel ischemia or necrosis, which occurred in Case 1, necessitating resection of ~30 cm of the ischemic small bowel segment. Other potential complications include gastrointestinal bleeding, volvulus, and rarely, perforation of the duplication cyst itself [9]. Additionally, advising patients to seek prompt medical attention when they experience unusual symptoms rather than attempting self-treatment at home which lead to severe complications as in Case 1, also plays a crucial role in treatment. Awareness of both the malignancy risk and the various complications associated with gastrointestinal duplication cysts plays a crucial role in guiding management decisions.

Surgical resection is often recommended for symptomatic or complicated cases, especially those involving the colon, regardless of whether patients with colonic duplication cysts are asymptomatic or symptomatic, surgical intervention is generally recommended to eliminate the risk of malignancy and ensure appropriate management [3, 8]. Moreover, postoperative pathological assessment is crucial for identifying potential malignant transformation of duplication cysts, which guides subsequent treatment strategies. Enteric duplication cysts must exhibit three defining characteristics: an epithelial lining resembling the mucosa of the gastrointestinal tract, a smooth muscle coat, and a shared wall with the gastrointestinal tract, forming an intimate attachment. The lining of duplication cysts can consist of various mucosal types, which include gastric, columnar, and squamous epithelium, predominantly derived from the gastrointestinal tract. [1, 12]. In the two cases under discussion, the surgical specimens underwent histopathological examination, which did not reveal any malignant features. Therefore, early diagnosis and timely intervention of complications arising from duplication cysts can lead to more favorable treatment outcomes.

Evaluating outcomes after surgical treatment is also a pertinent concern. Therefore, in our future work, a study assessing the post-operative status of patients would be necessary. However, due to the limited number of encountered cases, we currently lack sufficient data to conduct in-depth analytical research in Vietnam. Currently, in Vietnam in general as well as in the Mekong Delta region specifically, there is only one study evaluating the results of laparoscopic surgery for the treatment of duplication cyst in pediatric patients [15], with no research conducted on adults. Therefore, through the report of these two cases, the aim is to provide knowledge and a comprehensive overview of duplication cyst in adults, contributing to diagnosis and treatment. Additionally, there is a need for a larger study to analyse and draw more accurate conclusions in the context of Vietnam.

## Conclusion

Duplication cysts are rare congenital gastrointestinal anomalies that can present with varied symptoms or asymptomatic. There are no specific clinical signs, so diagnosis requires a combination of radiographic imaging techniques including ultrasound, CT scan, endoscopy, and especially (EUS-FNA) to evaluate the risk of malignancy before making treatment decisions. While the overall risk of malignancy in duplication cysts is 5%–6%, it is significantly higher (up to 67%) in colonic duplications. Potential complications include obstruction, bleeding, volvulus, and, rarely, perforation. Surgical resection is recommended for symptomatic and complicated cases, especially for asymptomatic colonic duplication cysts due to the high risk of malignancy, with some authors advocating surgical intervention as an appropriate treatment. However, there is currently a lack of standardized guidelines, particularly for adult cases of duplication cysts. An in-depth understanding of the clinical features, diagnostic approaches, malignancy risk, and treatment options for duplication cysts in adults is essential for effective diagnosis and management. This knowledge is crucial for the prompt identification and appropriate handling of duplication cysts in the adult population.

## Data Availability

Data are contained within the article. Data sharing does not apply to this article.

## References

[1] Radhakrishnan L , George J, Abraham LK. Right-sided colonic duplication cyst with a malignant twist in a young adult - a case report. J Gastrointest Cancer 2022;53:805–8. 10.1007/s12029-021-00671-5. 34279795

[2] Tiwari C , Shah H, Waghmare M, et al. Cysts of gastrointestinal origin in children: varied presentation. Pediatr Gastroenterol Hepatol Nutr 2017;20:94–9. 10.5223/pghn.2017.20.2.94. 28730133 PMC5517385

[3] Hsu H , Gueng MK, Tseng YH, et al. Adenocarcinoma arising from colonic duplication cyst with metastasis to omentum: a case report. J Clin Ultrasound 2011;39:41–3. 10.1002/jcu.20739. 20812340

[4] Rodríguez García P , Sánchez Pérez A, Romera Barba E, et al. Colonic duplication cyst in adult. Gastroenterol Hepatol 2020;43:360–1. 10.1016/j.gastrohep.2019.11.016. 32593469

[5] Singh JP , Rajdeo H, Bhuta K, et al. Gastric duplication cyst: two case reports and review of the literature. Case Rep Surg 2013;2013:605059. 23509656 10.1155/2013/605059 PMC3590563

[6] Pham L , et al. Intussusception characteristics and ultrasound guided pneumatic reduction a clinical experience in children less than 24 months old in Vietnam. Current Pediatric Research 2020;24:243–36.

[7] Shah A , Du J, Sun Y, et al. Dynamic change of intestinal duplication in an adult patient: a case report and literature review. Case Rep Med 2012;2012:297585. 22778749 10.1155/2012/297585 PMC3388337

[8] Liu R , Adler DG. Duplication cysts: diagnosis, management, and the role of endoscopic ultrasound. Endosc Ultrasound 2014;3:152–60. 10.4103/2303-9027.138783. 25184121 PMC4145475

[9] Gupta A , Chakaravarthi K, Pattnaik B, et al. Duplication cyst of ileum presenting as acute intestinal obstruction in an adult. BMJ Case Rep 2016;2016:bcr2016214775. 10.1136/bcr-2016-214775. PMC5073645 27758850

[10] Puligandla PS , Nguyen LT, St-Vil D, et al. Gastrointestinal duplications. J Pediatr Surg 2003;38:740–4. 10.1016/jpsu.2003.50197. 12720184

[11] Frering V , Velecela E, Fouque P, et al. Upper digestive duplications in adults. Ann Chir 1995;49:928–35. 8787320

[12] Sangüesa Nebot C , Llorens Salvador R, Carazo Palacios E, et al. Enteric duplication cysts in children: varied presentations, varied imaging findings. Insights Imaging 2018;9:1097–106. 10.1007/s13244-018-0660-z. 30311079 PMC6269332

[13] Shanmugalingam A , Duxbury H, Choi JDW, et al. An unusual case of colonic duplication cyst in an adult with dysplasia. J Surg Case Rep 2023;2023:rjad039. 10.1093/jscr/rjad039. 36824693 PMC9943052

[14] Inoue Y , Nakamura H. Adenocarcinoma arising in colonic duplication cysts with calcification: CT findings of two cases. Abdom Imaging 1998;23:135–7. 10.1007/s002619900305. 9516499

[15] Dinh AD , Pham DH. Outcomes of treatment of intestinal duplication cysts by laparoscopy at department of surgery of Vietnam national children's hospital between 2010-2020. Sci Res 2022;151:80–7.

